# Whole-Genome Sequencing of Mexican Strains of *Anaplasma marginale*: An Approach to the Causal Agent of Bovine Anaplasmosis

**DOI:** 10.1155/2020/5902029

**Published:** 2020-04-14

**Authors:** Fernando Martínez-Ocampo, Rosa Estela Quiroz-Castañeda, Itzel Amaro-Estrada, Edgar Dantán-González, Jesús Francisco Preciado de la Torre, Sergio Rodríguez-Camarillo

**Affiliations:** ^1^Laboratorio de Estudios Ecogenómicos, Centro de Investigación en Biotecnología, Universidad Autónoma del Estado de Morelos, C.P. 62209 Cuernavaca, Morelos, Mexico; ^2^Unidad de Anaplasmosis, Centro Nacional de Investigación Disciplinaria en Salud Animal e Inocuidad, INIFAP, C.P. 62574 Jiutepec, Morelos, Mexico

## Abstract

*Anaplasma marginale* is the main etiologic agent of bovine anaplasmosis, and it is extensively distributed worldwide. We have previously reported the first genome sequence of a Mexican strain of *A. marginale* (Mex-01-001-01). In this work, we report the genomic analysis of one strain from Hidalgo (MEX-14-010-01), one from Morelos (MEX-17-017-01), and two strains from Veracruz (MEX-30-184-02 and MEX-30-193-01). We found that the genome average size is 1.16-1.17 Mbp with a GC content close to 49.80%. The genomic comparison reveals that most of the *A. marginale* genomes are highly conserved and the phylogeny showed that Mexican strains cluster with Brazilian strains. The genomic information contained in the four draft genomes of *A. marginale* from Mexico will contribute to understanding the molecular landscape of this pathogen.

## 1. Introduction

Bovine anaplasmosis is an infectious, tick-borne disease caused mainly by *Anaplasma marginale*; typical signs include anemia, fever, abortion, weight loss, decreased milk production, jaundice, and potentially death. Although a sick bovine may recover when antibiotics are administered, it usually remains as a carrier for life, being a risk of infection for susceptible cattle. *Anaplasma marginale* is an obligate intracellular Gram-negative bacterium with a genetic composition that is highly diverse among geographical isolates [1]. Currently, there are no fully effective vaccines against bovine anaplasmosis; therefore, the economic losses due to the disease are present. Whole-genome sequencing (WGS) is an applicable tool for many pathogenic bacterial studies since 1995, when the first bacterial genomes were determined [2, 3]. Vaccine formulation became a hard task for pathogens as diverse as *Anaplasma marginale*, and almost all efforts have been directed toward Outer Membrane Proteins (Omp), Type IV Secretion System (T4SS), and Major Surface Proteins (Msp) [4–8]. Up to date, there are several genomes reported from *A. marginale*, but only one is from a Mexican strain [9]. New data could be useful for focusing in alternative antigens that induce specific and protective responses against bovine anaplasmosis. In this work, we present draft genomes from four *Anaplasma marginale* Mexican strains. In addition, a first approach for comparative analyses between them and Brazilian, Australian, and North American strains is shown. In order to advance in the identification of potential vaccine molecules, pathogenicity, transmission and infection mechanisms, and genetic diversity of *Anaplasma marginale*, further analyses are necessary.

## 2. Materials and Methods

### 2.1. Strain Origin

Each of the four Mexican strains were isolated from infected blood of animals from Atitalaquia, Hidalgo (MEX-14-010-01); Puente de Ixtla, Morelos (MEX-17-017-01); Tlapacoyan, Veracruz (MEX-30-184-02); and Veracruz, Veracruz (MEX-30-193-01). The infected blood was collected and kept at -80°C until its use.

### 2.2. Genome Sequencing, Assembly, and Annotation

We used 200 *μ*l of bovine blood for each isolate to extract genomic DNA using the UltraClean DNA BloodSpin kit (Mo Bio Laboratories). The library preparation was performed by the University of Arizona Genetics Core, using a DNA TruSeq library construction kit (Illumina). Two micrograms of genomic DNA for each isolate was sequenced with MiSeq platform (Illumina). The NextSeq instrument from Illumina uses sequencing-by-synthesis (SBS) chemistry. The Illumina adapter sequences were removed from paired-end reads using ILLUMINACLIP trimming step of the Trimmomatic (version 0.36) program with default settings [10]. Low-quality bases were removed using the dynamictrim algorithm of SolexaQA++ (version 3.1.7.1) suite [11] with a Phred quality score *Q* < 13. The resulting paired-end reads were *de novo* assembled using the SPAdes (version 3.11.1) program [12] with the following options: (i) only runs assembly module (--only-assembler), (ii) reduce number of mismatches (--careful), and (iii) *k*-mer lengths between 21 and 127. Based on the G+C content of each contig assembled using a Python script (https://github.com/FernandoMtzMx/GC_content_MultiFasta) (*A. marginale* genomes reported in databases have a G+C content between 46 and 52%), contigs of four Mexican strains were differentiated from contigs that belong to other organisms (i.e., bovine genomes). Also, we aligned the sequences of each contig assembled with the nucleotide collection (nr/nt) database and *Anaplasma marginale* as the organism name using BLASTN suite [13]. Contigs with an alignment coverage higher than 50% and an identity higher than 70% belong to *A. marginale* genomes were considered “reasonably good” alignments [14]. The features of four draft genomes were evaluated using the QUAST (version 4.6.2) program [15].

The draft genomes of four Mexican strains were annotated automatically using the RAST (version 2.0) server [16], and the 16S rRNA gene sequences were obtained using the RNAmmer (version 1.2) server [17].

### 2.3. Genomic Comparison

The Blast Ring Image Generator (BRIG) (v0.95) program [18] was used to determine the genome comparison between the Mexican *A. marginale* strains and six strains from Australia, Brazil, and the United States. The circular comparative genomic map was constructed by BRIG using the GenBank files (gbk format) with standard default parameters and NCBI local blast-2.9.0+ suite.

### 2.4. Phylogenetic Analysis

The 16S rRNA, *gyrA*, *gyrB*, *groEL*, and *rpoB* sequences of housekeeping genes were obtained from the genomes of MEX-01-001-01 (Aguascalientes, Aguascalientes), MEX-14-010-01 (Atitalaquia, Hidalgo), MEX-17-017-01 (Puente de Ixtla, Morelos), MEX-30-184-02 (Tlapacoyan, Veracruz), and MEX-30-193-01 (Veracruz, Veracruz). The gene sequence datasets of Mexican strains were compared to 13 downloaded gene sequence datasets of *A. marginale*, *A. centrale*, *A. ovis*, *A. phagocytophilum*, and *Ehrlichia canis* and *E. ruminantium* (as outgroup), which were obtained from the GenBank database (https://www.ncbi.nlm.nih.gov/) using the nucleotide BLAST suite (https://blast.ncbi.nlm.nih.gov/Blast.cgi). Multiple alignments between all gene sequence datasets were made using the MUSCLE (v3.8.31) program [19]. Alignment sequences per genome were concatenated using a Python script. The jModelTest (v2.1.10) program [20] was used to select the best model of nucleotide substitution using the Akaike information criterion (AIC). Phylogenetic tree was inferred based on a maximum likelihood method using the PhyML (v3.1) program [21] with 1000 bootstrap replicates. The phylogenetic tree was visualized and edited using the FigTree (v1.4.3) program (http://tree.bio.ed.ac.uk/software/figtree/).

### 2.5. Genome Synteny Analysis

The presence of large-scale evolutionary events, such as rearrangement and inversion of genomic segments, was detected by aligning the ordered contigs of the four Mexican strains and the genomes of Florida, Dawn, Gypsy Plains, Jaboticabal and Palmeira strains (GenBank accession numbers CP001079.1, CP006847.1, CP006846.1, CP023731.1, and CP023730.1, respectively), against the reference genome of *A. marginale* St. Maries (GenBank accession number CP000030.1), when using the “Align with progressiveMauve” algorithm of the Mauve program (v2.4.0) [22].

## 3. Results

### 3.1. Data Availability

The Whole Genome Shotgun project has been deposited at DDBJ/ENA/GenBank under the accession VTSO00000000 (MEX-14-010-01), VTCX00000000 (MEX-17-017-01), VTCY00000000 (MEX-30-184-02), and VTCZ00000000 (MEX-30-193-01).

### 3.2. Genome Features

We obtained four random datasets of 1,418,888 (MEX-14-010-01); 567,482 (MEX-17-017-01); 671,050 (MEX-30-184-02); and 940,598 (MEX-30-193-01) paired-end reads of 300 bp which were reported in the GenBank database. The number of reads obtained after Trimmomatic and dynamictrim analyses are as follows: MEX-30-184-02, 669,396; MEX-30-193-01, 940,392; MEX-17-017-01, 567,434; and MEX-14-010-01, 1,418,550; the assembly coverage is 27X, 154X, 58X, and 22X, respectively.

The GC content for MEX-14-010-01, MEX-17-017-01, and MEX-30-184-02 strains is 49.79% and 49.80% for MEX-30-193-01. The percentage of GC values for Mexican strains is very similar to that for the reference strain *A. marginale* St. Maries (49.80%). Other genomic characteristics of each strain are shown in Table 1.

According to the RNAmmer server, all 16S rRNA Mexican strains have a length of 1,491 bp, which have 100% alignment coverage, and between 99 and 100% are identified with the 16S rRNA gene sequence of *A. marginale* strain St. Maries (GenBank accession CP000030). The number of rRNAs and tRNAs in the four strains is 3 and 37, respectively. The number of protein-coding genes for MEX-14-010-01, MEX-17-017-01, MEX-30-184-02, and MEX-30-193-01 is 1150, 1163, 1165, and 1138, respectively. In Table 2, the information derived from the SEED subsystem of the RAST server for each strain is shown.

### 3.3. Genomic Comparison and Phylogeny

**Figure 1:**
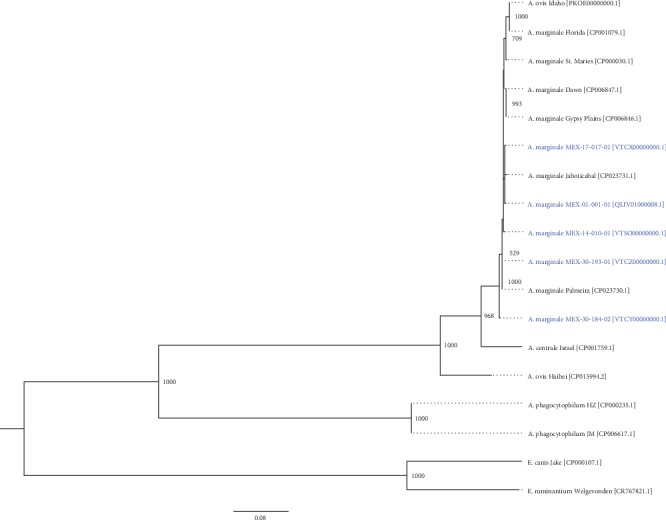
Phylogeny obtained based on the concatenated sequences of five housekeeping genes (16S rRNA, *gyA*, *gyrB*, *groEL*, and *rpoB*). The tree shows that Mexican strains of the *A. marginale* cluster with Brazilian strains, specifically, MEX-01-001-01 and MEX-17-017-01 strains that cluster with *A. marginale* Jaboticabal. The phylogenetic tree was obtained using the PhyML (v3.1) program with the maximum likelihood method and 1000 bootstrap replicates. GenBank accession numbers of the genomes are shown in square brackets. Bootstrap values are displayed in the nodes.

**Figure 2:**
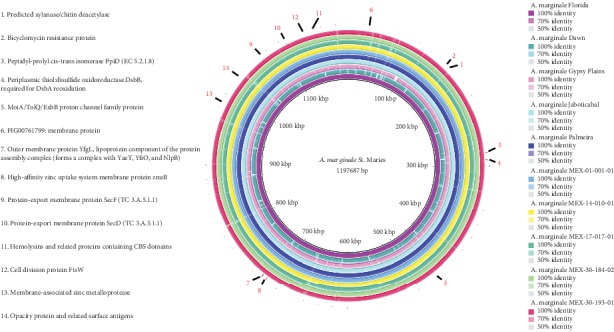
Circular comparative genomic map between five Mexican strains with six strains of *A. marginale* from Australia, Brazil, and the United States. The innermost ring (black) represents the *A. marginale* St. Maries genome (length of 1,197,687 bp). Rings 2-11 represent the *A. marginale* genomes of Florida, Dawn, Gypsy Plains, Jaboticabal, Palmeira, MEX-01-001-01, MEX-14-010-01, MEX-17-017-01, MEX-30-184-02, and MEX-30-193-01 strains, respectively. The outermost ring highlights 10 genes (red arrows and numbers) that are highly conserved in the genomes. The annotated function on the RAST server of the 10 proteins is shown on the left side.

We compared the four Mexican draft genomes of *A. marginale* with Brazilian, Australian, and North American strains. In Figure 1, the comparative genomics is shown. Although most of the genomes are highly conserved, the Dawn and Gypsy Plains strains showed some differences from the Mexican, North American, and Brazilian strains. We randomly selected fourteen ORFs found in the genomic annotation predicted as membrane proteins, and then, we located them in the genomes; as observed, most of these proteins are conserved in all genomes (Figure 2).

### 3.4. Genome Synteny Analysis

**Figure 3:**
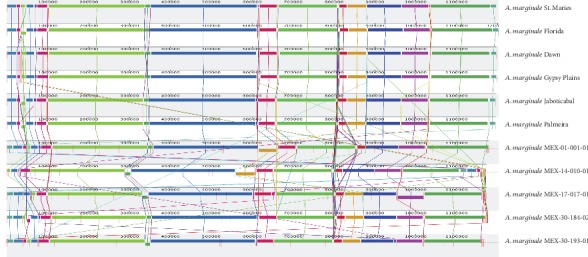
Genome synteny of *A. marginale* genomes of Australian, Brazilian, Mexican, and North American strains. In general, a highly conserved structure is observed in all genomes, with the exception of Mexican strains that show rearrangements and inversions along the genome sequences.

The genome synteny of 11 *A. marginale* genomes of Australian, Brazilian, Mexican, and North American strains shows that the first 100,000 bases have a rearrangement of several small fragments (Figure 3). In addition, the genome synteny of *A. marginale* shows that the Australian, Brazilian, and North American strains have a highly conserved genome structure, while the genomes of Mexican strains show some rearrangement and inversion of genomic segments (Figure 3). In general, the structure of *A. marginale* genomes shares a high percentage of coverage and is widely conserved in different geographical regions of the world.

## 4. Discussion

So far, only one draft genome of a Mexican strain of *A. marginale* has been reported [9]. In this work, we present the genomic information of other four strains: MEX-14-010-01, MEX-17-017-01, MEX-30-184-02, and MEX-30-193-01.

The genomic analysis reveals that their size (ranging from 1,167,111 bp to 1,176,681 bp) and a GC content (about 49.79%) are very similar to other *A. marginale* strains reported in GenBank such as the reference genome of the St. Maries strain, with a genome size of 1,197,690 bp and a GC content of 49.80%.

The number of Genes and CDS is very similar in the four strains. In fact, in the genome annotation, using the different subsystem classification of RAST server, we identified genes related to cell wall and capsule, virulence, disease and defense, membrane transport, and protein and DNA metabolism, among others. In the virulence, disease, and defense categories, we found genes associated with the cobalt-zinc-cadmium resistance, fluoroquinolone resistance, cooper homeostasis, and beta lactamase. Also, we identified genes of *Mycobacterium* virulence operon involved in protein synthesis (SSU and LSU ribosomal proteins) and *Mycobacterium* virulence operon involved in DNA transcription. *Mycobacterium* operon is present in several species, including *Mycobacterium tuberculosis*, *Streptococcus pneumoniae*, *Bartonella bovis*, and *Streptococcus suis*, among other animal and plant pathogens [23–25].

In the stress response category, we found genes associated with oxidative stress, cold shock, heat shock, periplasmic stress response, and detoxification. For most of the obligate intracellular bacteria, the presence of peptidoglycan is not necessarily needed to maintain the integrity of the bacterial cell. In *A. marginale*, there are no reports of the analysis or isolation of its peptidoglycan [26]; however, we identified genes associated with the cell wall and capsule, specifically with the peptidoglycan biosynthesis. An interesting feature of *A. marginale* genomes is the role of the genes that we found in nitrogen metabolism. In alphaproteobacteria, the role of nitrogen metabolism may be essential for full virulence [27].

The phylogeny analysis indicates that Mexican strains are more related to Brazilian strains than to North American ones. The genomic comparison of the strains reveals the high percent of identity between *A. marginale* genomes as observed in the genome synteny analysis, where most of the strains are highly conserved in its structure and the Mexican strains have some rearrangements and inversions in certain genomic sequences.

The report of four draft genomes of *A. marginale* found in Mexico represents a first approach to unveil information that could help to develop new strategies for the design of vaccines against bovine anaplasmosis and new diagnostic methods. Still, more genomic analyses are needed to complete the molecular landscape of this pathogen.

## 5. Conclusions

We present here, the genomic report and analyses of four Mexican strains of *A. marginale*, the causal agent of bovine anaplasmosis. So far, only one genome of a Mexican strain has been reported; with this contribution, we compare our results with information of strains from the USA, Brazil, and Australia and provide more information of this pathogen.

## Figures and Tables

**Table 1:** Genomic statistics of the four draft genomes of Mexican strains.

Strain	MEX-14-010-01	MEX-17-017-01	MEX-30-184-02	MEX-30-193-01
Genome size (bp)	1,172,327	1,172,716	1,176,681	1,167,111
Contigs	46	41	40	41
N_50_ length (bp)	85,147	65,203	61,328	65,442
Coverage	~22X	~58X	~27X	~154X
G+C content	49.79	49.79	49.79	49.80
Genes	1,190	1,203	1,205	1,178
CDS	1,150	1,163	1,165	1,138

**Table 2:** RAST annotation categories of Mexican strain genomes.

Strain	MEX-14-010-01	MEX-17-017-01	MEX-30-184-02	MEX-30-193-01
Cofactors, vitamins, prosthetic groups, pigments	124	128	124	120
Cell wall and capsule	37	37	37	37
Virulence, disease, and defense	17	18	18	17
Potassium metabolism	3	3	3	3
Miscellaneous	4	4	4	4
Phages, prophages, transposable elements, plasmids	1	1	1	1
Membrane transport	13	13	14	13
Iron acquisition and metabolism	3	3	3	3
RNA metabolism	52	50	52	50
Nucleosides and nucleotides	42	46	42	43
Protein metabolism	185	189	188	185
Cell division and cell cycle	24	24	26	24
Regulation and cell signaling	1	1	1	1
DNA metabolism	51	52	46	49
Fatty acids, lipids, and isoprenoids	54	56	54	56
Nitrogen metabolism	5	5	5	5
Dormancy and sporulation	1	1	1	1
Respiration	61	61	60	69
Stress response	26	26	26	26
Amino acids and derivatives	51	51	51	51
Sulfur metabolism	4	4	4	4
Phosphorus metabolism	9	9	9	9
Carbohydrates	41	43	45	43

## Data Availability

The Whole Genome Shotgun project has been deposited at DDBJ/ENA/GenBank under the accession: VTSO00000000 (MEX-14-010-01), VTCX00000000 (MEX-17-017-01), VTCY00000000 (MEX-30-184-02) and VTCZ00000000 (MEX-30-193-01).

## Additional Points

*Reference Numbers for Data Available in GenBank*. MEX-30-184-02 *Anaplasma marginale* (GenBank ID: VTCY00000000); MEX-30-193-01 *Anaplasma marginale* (GenBank ID:VTCZ00000000); MEX-17-017-01 *Anaplasma marginale* (GenBank ID:VTCX00000000); MEX-14-010-01 *Anaplasma marginale* (GenBank ID:VTSO00000000).

## References

[1] Quiroz-Castañeda R. E., Amaro-Estrada I., Rodríguez-Camarillo S. D. (2016). Anaplasma marginale: diversity, virulence, and vaccine landscape through a genomics approach. *BioMed Research International*.

[2] Fleischmann R., Adams M., White O. (1995). Whole-genome random sequencing and assembly of Haemophilus influenzae Rd. *Science*.

[3] Fraser C. M., Gocayne J. D., White O. (1995). The minimal gene complement of Mycoplasma genitalium. *Science*.

[4] Agnes J. T., Brayton K. A., LaFollett M., Norimine J., Brown W. C., Palmer G. H. (2011). Identification of Anaplasma marginale outer membrane protein antigens conserved between A. marginale sensu stricto strains and the live A. marginale subsp. centrale vaccine. *Infection and Immunity*.

[5] Brown W. C., Zhu D., Shkap V. (1998). The repertoire of Anaplasma marginale antigens recognized by CD4(+) T-lymphocyte clones from protectively immunized cattle is diverse and includes major surface protein 2 (MSP-2) and MSP-3. *Infection and Immunity*.

[6] Ducken D. R., Brown W. C., Alperin D. C. (2015). Subdominant outer membrane antigens in Anaplasma marginale: conservation, antigenicity, and protective capacity using recombinant protein. *PLoS One*.

[7] Lopez J. E., Palmer G. H., Brayton K. A., Dark M. J., Leach S. E., Brown W. C. (2007). Immunogenicity of Anaplasma marginale type IV secretion system proteins in a protective outer membrane vaccine. *Infection and Immunity*.

[8] Zhao L., Mahony D., Cavallaro A. S. (2016). Immunogenicity of outer membrane proteins VirB9-1 and VirB9-2, a novel nanovaccine against Anaplasma marginale. *PLoS One*.

[9] Quiroz Castañeda R. E., Amaro-Estrada I., Martínez-Ocampo F. (2018). Draft genome sequence of Anaplasma marginale strain Mex-01-001-01, a Mexican strain that causes bovine anaplasmosis. *Microbiology Resource Announcements*.

[10] Bolger A. M., Lohse M., Usadel B. (2014). Trimmomatic: a flexible trimmer for Illumina sequence data. *Bioinformatics*.

[11] Cox M. P., Peterson D. A., Biggs P. J. (2010). SolexaQA: at-a-glance quality assessment of Illumina second-generation sequencing data. *BMC Bioinformatics*.

[12] Bankevich A., Nurk S., Antipov D. (2012). SPAdes: a new genome assembly algorithm and its applications to single-cell sequencing. *Journal of Computational Biology*.

[13] Altschul S. F., Gish W., Miller W., Myers E. W., Lipman D. J. (1990). Basic local alignment search tool. *Journal of Molecular Biology*.

[14] Sherman R. M., Forman J., Antonescu V. (2019). Assembly of a pan-genome from deep sequencing of 910 humans of African descent. *Nature Genetics*.

[15] Gurevich A., Saveliev V., Vyahhi N., Tesler G. (2013). QUAST: quality assessment tool for genome assemblies. *Bioinformatics*.

[16] Aziz R. K., Bartels D., Best A. A. (2008). The RAST Server: rapid annotations using subsystems technology. *BMC Genomics*.

[17] Lagesen K., Hallin P., Rødland E. A., Staerfeldt H.-H., Rognes T., Ussery D. W. (2007). RNAmmer: consistent and rapid annotation of ribosomal RNA genes. *Nucleic Acids Research*.

[18] Alikhan N.-F., Petty N. K., Ben Zakour N. L., Beatson S. A. (2011). BLAST Ring Image Generator (BRIG): simple prokaryote genome comparisons. *BMC Genomics*.

[19] Edgar R. C. (2004). MUSCLE: multiple sequence alignment with high accuracy and high throughput. *Nucleic Acids Research*.

[20] Darriba D., Taboada G. L., Doallo R., Posada D. (2012). jModelTest 2: more models, new heuristics and parallel computing. *Nature Methods*.

[21] Guindon S., Gascuel O. (2003). A simple, fast, and accurate algorithm to estimate large phylogenies by maximum likelihood. *Systematic Biology*.

[22] Darling A. C., Mau B., Blattner F. R., Perna N. T. (2004). Mauve: multiple alignment of conserved genomic sequence with rearrangements. *Genome Research*.

[23] Gao L.-Y., Pak M., Kish R., Kajihara K., Brown E. J. (2006). A mycobacterial operon essential for virulence in vivo and invasion and intracellular persistence in macrophages. *Infection and Immunity*.

[24] Sun Y., Veseli I. A., Vaillancourt K., Frenette M., Grenier D., Pombert J. F. (2019). The bacteriocin from the prophylactic candidate Streptococcus suis 90-1330 is widely distributed across S. suis isolates and appears encoded in an integrative and conjugative element. *PLoS One*.

[25] Tay S. T., Kho K. L., Lye S. F., Ngeow Y. F. (2018). Phylogeny and putative virulence gene analysis of Bartonella bovis. *The Journal of Veterinary Medical Science*.

[26] Otten C., Brilli M., Vollmer W., Viollier P. H., Salje J. (2018). Peptidoglycan in obligate intracellular bacteria. *Molecular Microbiology*.

[27] Ronneau S., Moussa S., Barbier T. (2016). Brucella, nitrogen and virulence. *Critical Reviews in Microbiology*.

